# How can I protect my child?: A Delphi guidelines study on parenting to prevent body image and eating problems in young children

**DOI:** 10.1186/2050-2974-2-S1-O30

**Published:** 2014-11-24

**Authors:** Laura Hart, Stephanie Damiano, Susan Paxton, Anthony Jorm

**Affiliations:** 1 La Trobe University, Melbourne, Australia; 2 University of Melbourne, Melbourne, Australia

## 

The foundations for positive body image and healthy eating patterns are developed in early childhood. Educating parents about how they can promote these in their young children could help prevent body dissatisfaction and disordered eating, lower risk for obesity and improve child wellbeing. The purpose of this study was to produce guidelines for parents of 2-6 year olds, on how they can prevent or reduce the risk that their child will develop eating or body image problems. This involved establishing expert consensus through an online Delphi study with a panel of research or clinical experts in the fields of eating disorders, body image, obesity or parenting. Experts were asked to rate items, describing potential parenting strategies, according to how important they believed each was for parents. Stringent criteria were used for accepting items as guidelines and three rounds were completed before consensus was reached. Twenty-two panel members with a wide range of expertise rated a total of 338 items, from which a set of guidelines with a high level of expert consensus was established. A guideline document was developed and will be used to inform prevention interventions for parents of pre-school children.

This abstract was presented in the **Parental Roles in Prevention and Support** stream of the 2014 ANZAED Conference.

